# Demographic factors associated with dietary supplement prescriptions filled by United States Military Service Members 2005–2013

**DOI:** 10.1186/s12906-017-1590-x

**Published:** 2017-01-31

**Authors:** Joseph J. Knapik, Rosenie T. Jean, Krista G. Austin, Ryan A. Steelman, Emily K. Farina, Harris R. Lieberman

**Affiliations:** 1 US Army Research Institute of Environmental Medicine, Natick, MA USA; 2 US Army Public Health Center, Aberdeen Proving Ground, MD USA; 3 Oak Ridge Institute for Science and Education, Belcamp, MD USA; 4 Office of The US Army Surgeon’s General Pharmacovigilance Center, Falls Church, VA USA; 5 Defense Health Agency, Falls Church, VA USA; 6 Research Physiologist, (USARIEM), 10 General Greene Ave, Natick, MA 01760 USA

**Keywords:** Multivitamins, Vitamins, Minerals, Iron, Zinc, Replacement preparations, Sodium/potassium compounds, Antacids, Absorbents

## Abstract

**Background:**

Dietary supplements (DSs) can be purchased over-the-counter but may also be prescribed by medical personnel for specific therapeutic reasons. Few studies have examined this latter source of DSs despite the fact that 79% of physicians and 82% of nurses have recommended DSs to their patients. This investigation examined demographic factors associated with temporal trends in oral DS prescriptions filled by all United States (US) service members (SMs) from 2005 to 2013 (*n* = 1,427,080 ± 22,139, mean ± standard deviation per year).

**Methods:**

The Food and Drug Administration National Drug Code database and the formularies of the US Defense Health Agency’s Pharmacoeconomic Center were queried to identify DSs available to SMs. The number of these DS prescriptions filled by all SMs from 2005 through 2013 was then obtained from the US Department of Defense Pharmacy Data Transaction System. Data were grouped by American Hospital Formulary System (AHFS) pharmacologic-therapeutic classifications and examined over time. Denominators (number of SMs each year) were obtained from the Defense Health Agency.

**Results:**

Major findings included 1) generally greater prevalence of prescriptions filled by women and older SMs for most AHFS categories; 2) a temporal decline in total prescriptions filled by Marine Corps personnel accounted for by a decline in the prevalence of zinc preparations filled by younger male Marines; 3) a temporal decline in the prevalence of iron preparations filled by women; 4) a temporal increase in the prevalence of prescriptions for replacement preparations filled by women accounted for largely by more prescriptions for calcium compounds; and 5) a temporal decline in the prevalence of prescriptions filled for cathartics/laxatives in older SMs accounted for largely by a decline in prescriptions for sodium/potassium compounds.

**Conclusions:**

These temporal trends may be associated with the greater health care utilization of women and older SMs as well as the perceptions of prescribers and/or patients on appropriate roles of these substances in medicine and public health.

## Background

Dietary supplements (DSs) are commercially available products that are consumed as an addition to the usual diet and include vitamins, minerals, herbs (botanicals), amino acids and a variety of other substances [1]. Surveys of health care providers found that 79% of physicians, 82% of nurses, and 97% of dietitians had recommended DSs to their patients [2, 3]. Numerous investigations have quantified the prevalence of self-prescribed, over-the-counter DS use among civilians [4–7] and military personnel [8–11]. However, in addition to purchasing DSs over-the-counter, military service members (SMs) can obtain prescriptions for DSs from their medical care providers and fill those prescriptions in the military health care pharmacy system. Information on filled prescriptions is documented by the US Department of Defense Pharmacy Data Transaction System (PDTS) along with specific demographic information. This provides an opportunity to examine DS prescription prevalence and temporal trends in relation to the demographic characteristics of the SM population.

A few studies have examined prescribed DS use among civilians, but these studies are based on self-reports rather than information obtained from medical records [12–14], and only one study [12] has examined the demographics of users. The US Department of Defense PDTS captures all medications dispensed at military treatment facilities and retail pharmacies. A few previous studies used PDTS data to examine trends in some DS prescriptions to all personnel (military and dependents) eligible for care in military medical treatment facilities from 2007 to 2011 [15–17] and we have described the overall temporal trends in DS prescriptions filled by SMs from 2005 to 2013 [18]. In this paper, we describe temporal trends in DS prescriptions in relation to specific demographic characteristics including military service, sex, and age.

## Methods

This was a descriptive study designed to identify demographic factors associated with patterns of oral DS prescriptions filled by US military SMs from 2005 through 2013. SMs included the entire population of active duty personnel in the Army, Navy, Marine Corps, Air Force, and Coast Guard. DSs were defined based on the Dietary Supplement Health and Education Act of 1994 as “…a product (other than tobacco) intended to supplement the diet that bears or contains one or more of the following dietary ingredients: a) a vitamin; b) a mineral; c) an herb or other botanical; d) an amino acid; e) a dietary substance for use by man to supplement the diet by increasing total dietary intake; or f) a concentrate, metabolite, constituent, extract or combination of any ingredient in clause a to e” [1]. The study was approved by the Institutional Review Board of the US Army Research Institute of Environmental Medicine.

To identify DSs available for prescription to SMs two databases were queried: the Food and Drug Administration National Drug Code (NDC) database [19] and the US Defense Health Agency’s Pharmacoeconomic Center (PEC) database [20]. The NDC database was obtained in November 2013 and contained 65,536 listed substances. For the PEC database, the basic and extended core formularies were examined using the search engine at the PEC website [20]. The following search terms were used to identify substances classified by First Data Bank as Generic Class 3 (GC3) categories of drugs which could also be DSs: vitamins, minerals, protein and amino acids, herbs and botanical ingredients, fish oil, creatine, joint support, digestive, and DSs. The GC3 system utilizes 3 characters (alpha, numeric, and alpha) to represent the organ system, pharmacological class, and specific therapeutic class. A total of 34,901 listed substances were identified and the corresponding NDC numbers, GC3 numbers, and generic names were provided by a pharmacist from the Defense Health Agency Pharmacy Operations Division. Two investigators independently examined the two databases to identify DSs and subsequently met to resolve any discrepancies. Only substances listed as orally consumed were considered and all substances listed as “unapproved homeopathics” were excluded. After eliminating overlapping substances in the two databases, the process resulted in the identification of 25,291 unique oral DS substances with distinctive NDCs and GC3s codes (A1B, B3K, C1B, C1D, C1F, C1H, C1P, C1W, C3B, C3C, C3M, C5B, C5F, C5G, C5H, C5U, C6A, C6B, C6C, C6D, C6E, C6F, C6G, C6H, C6I, C6L, C6M, C6N, C6P, C6Q, C6R, C6T, C6Z, C8E, D4B, D4N, D6S, E0A, E0G, M4B, M4O, P4D, U5B, U6W, and Z1E).

Once DSs were identified, data were extracted from PDTS records. NDCs were used as the unit of identification to obtain substances dispensed to a SM between 2005 through 2013. The PDTS database was queried to obtain the number of SMs filling one or more prescriptions. Because of the large number of NDCs, the list was further reduced by grouping the NDC codes according to their generic names, as listed in the database (e.g., aluminum hydroxide, cholecalciferol, arginine, calcium, psyllium). In 53 cases there were no generic names listed but 48 of these records were some form of Vitamin E and so these were given this generic name. The other 5 substances were not prescribed in the period examined. Using generic names resulted in 1,711 categories. Of the 1,711 generic categories, 488 had at least one SM receiving a prescription in the study period. For each of the 488 generic categories, data were obtained from the PDTS on the military service, sex, and age of SM who obtained the DS prescriptions.

A pharmacist placed the 488 generic substances into the American Hospital Formulary System (AHFS) Pharmacologic-Therapeutic classifications [21]. After this, 5 AHFS Pharmacologic-Therapeutic classifications were combined because they were: 1) subcategories of higher tier AHFS codes; 2) had a small number of prescriptions in the particular therapeutic classes; and/or 3) had very similar therapeutic classifications. One group of pharmacologic-therapeutic classifications that was combined were 28:20.00 (Anorexigenic Agents and Respiratory and Cerebral Simulants), 28:20.32 (Respiratory and Central Nervous System Stimulants), and 28:20.92 (Anorexigenic Agents and Stimulants, Miscellaneous). The other pharmacologic-therapeutic classifications that were combined were 92:00.00 (Miscellaneous Therapeutic Agents) and 92:92.00 (Other Miscellaneous Therapeutic Agents).

Prevalence of prescriptions filled for various AHFS categories was calculated as: ∑ of SMs filling one or more prescription in a particular year divided by ∑ of SMs for the year times 1,000 (SMs with one or more prescriptions/1,000 SMs). The Armed Forces Health Surveillance Branch of the Defense Health Agency provided denominators (∑ SMs for each year) for each demographic factor (military service, sex, and age). To examine trends, data were graphed by year for each AHFS category and demographic factor. Descriptive statistics were not necessary for DS data since the data included all SMs and the point values on the graphs represent population values (i.e., values are not from a sample for which statistics would be required).

## Results

Table 1 presents the number of SMs filling one or more oral DS prescriptions by AHFS category and demographics. During the 9-year surveillance period, 1,448,750 SMs filled prescriptions, a mean ± standard deviation of 160,972 ± 5,128 SMs per year. The mean ± standard deviation yearly number of SMs was 1,427,080 ± 22,139 so that 11.3% (160,972/1,427,080) of all SMs filled an oral DS prescription each year during the study period. Five AHFS pharmacologic-therapeutic categories accounted for <0.2% of all individuals filling prescriptions and were not considered further. These were 1) anorexigenic, respiratory, and central nervous system stimulants; 2) caloric agents; 3) gastrointestinal drugs; 4) Vitamin A; and 5) therapeutic agents.

**Table 1:** Number of service members filling DS prescriptions by AHFS category and demographics, 2005–2013

Variable	Strata	200404 Iron Prep	282000, 282032 & 282093 Anorexigenic, Respiratory & CNS Stimulants	401200 Replace-ment Prep	402000 Caloric Agents	560400 Antacids & Absorbents	561200 Cathartics & Laxatives	569200 GI Drugs, Misc	880400 Vit A	880800 Vit B Complex	881200 Vit C	881600 Vit D	882000 Vit E	882800 Multivit Prep	920000 & 929200 Therapeutic & Misc Therapeutic Agents
Military Service	Army	67,714	17	102,499	300	41,812	86,321	7	110	57,252	60,408	38,874	2,512	179,149	1,468
	Navy	39,190	5	70,814	5	17,779	47,290	1	32	41,547	26,106	14,283	1,956	100,821	180
	Marine Corps	8,830	2	41,620	3	5,106	25,390	5	12	17,214	6,013	4,684	484	31,871	45
	Air Force	36,997	2	26,835	3	5,365	26,209	0	18	28,383	4,839	19,109	122	126,076	435
	Coast Guard	1,823	1	2,342	0	635	4,263	0	4	1,824	611	1,352	532	10,860	22
	Unknown	920	0	2,017	0	192	1,190	0	1	729	184	1,623	76	3,419	6
Gender	Men	18,943	22	100,398	294	37,350	129,771	12	114	89,746	62,311	49,309	3,281	44,776	1,729
	Women	136,529	5	145,726	17	33,539	60,890	1	63	57,203	35,849	30,616	2,401	407,417	427
	Unknown	2	0	3	0	0	2	0	0	0	1	0	0	3	0
Age	<25 Years	66,401	7	139,784	120	44,419	65,183	7	44	57,426	45,911	11,266	1,838	194,169	349
	25–34	55,258	11	51,264	132	14,930	55,253	6	64	51,521	35,214	24,674	1,373	190,783	647
	35–44	25,922	8	32,351	51	7,877	42,828	0	47	26,793	13,383	27,473	1,441	57,365	683
	>44	7,893	1	22,728	8	3,663	27,399	0	22	11,209	3,653	16,512	1,030	9,879	477

*Abbreviations*: *DS* dietary supplements, *Prep* preparations, *CNS* central nervous system, *GI* gastrointestinal, *Misc* miscellaneous, *Vit* vitamin, *Multivit* multivitamin

### Temporal changes by military service

**Fig. 1:**
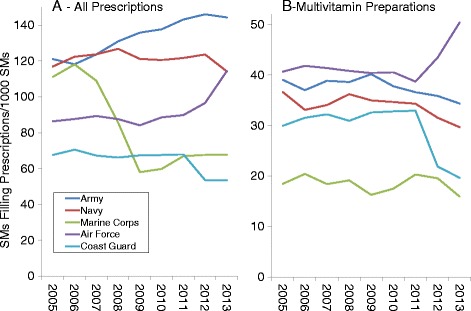
Prevalence of Prescriptions Filled for All Prescriptions (**a**) and Multivitamin Preparations (**b**) by Military Service, 2005–2013

**Fig. 2:**
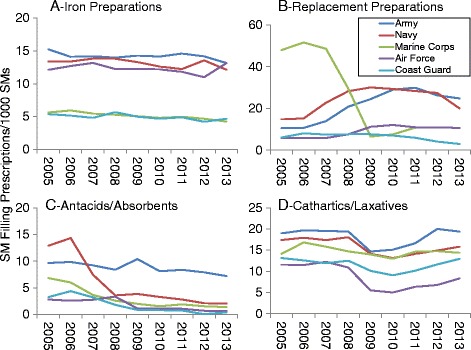
Prevalence of Prescriptions Filled for Iron Preparations (**a**), Replacement Preparations (**b**), Antacids/Absorbents (**c**) and Cathartics/Laxatives (**d**) by Military Service, 2005–2013

**Fig. 3:**
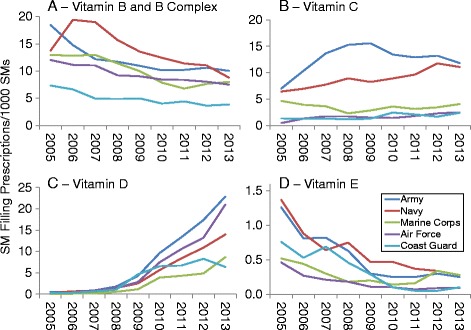
Prevalence of Prescriptions Filled for Vitamin B and B Complex (**a**), Vitamin C (**b**), Vitamin D (**c**), and Vitamin E (**d**) by Military Service, 2005–2013

Figures 1, 2 and 3 present DS prevalence by AHFS categories, year, and military service. Prevalence for all prescribed DSs (Fig. 1a) increased over time for Army and Air Force personnel, while for Marine Corps personnel prevalence declined by about half between 2006 and 2009. For multivitamin preparations (Fig. 1b) the Army and Navy showed very slight declines over time and after 2011, prevalence for the Air Force rose while that of the Coast Guard declined. Iron preparation prevalence (Fig. 2a) was much lower for Coast Guard and Marine personnel than for the other services but all services showed little change over time. For replacement preparations (Fig. 2b), Marine Corps personnel demonstrated a 7.5-fold decline in prevalence from 2007 to 2009 while Army, Navy, and to a lesser extent, Air Force personnel showed an increase between 2006 and 2009–2010. Antacid/absorbent prevalence (Fig. 2c) generally declined in all military services over the study period, with the largest being a 4-fold decline among Navy personnel between 2006 and 2008. Prevalence of cathartic/laxative prescriptions (Fig. 2d) generally declined in most services in 2009, but rose to earlier levels by 2012 in all services except the Air Force.

Vitamin B and B complex (Fig. 3a) prevalence generally declined for all military services over the study period. Vitamin C prevalence (Fig. 3b) was highest for Army and Navy personnel and all services except the Marine Corps showed increased prevalence during the study period. Vitamin D prevalence (Fig. 3c) increased in all services, while Vitamin E prevalence (Fig. 3d) declined in all services during the study period.

### Temporal changes by sex

**Fig. 4:**
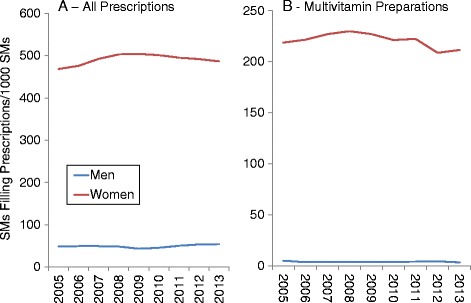
Prevalence of Prescriptions Filled for All Prescriptions (**a**) and Multivitamin Preparations (**b**) by Sex, 2005–2013

**Fig. 5:**
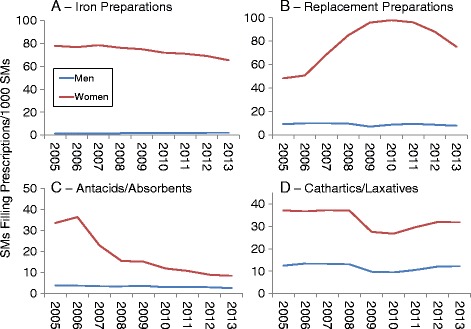
Prevalence of Prescriptions Filled for Iron Preparations (**a**), Replacement Preparations (**b**), Antacids/Absorbents (**c**) and Cathartics/Laxatives (**d**) by Sex, 2005–2013

**Fig. 6:**
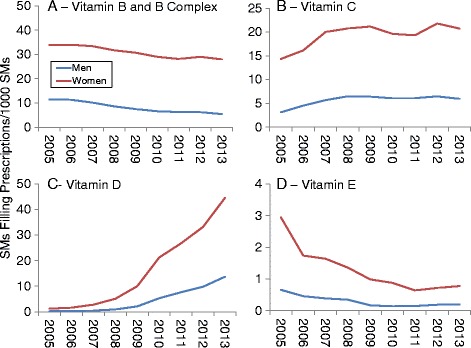
Prevalence of Prescriptions Filled for Vitamin B and B Complex (**a**), Vitamin C (**b**), Vitamin D (**c**), and Vitamin E (**d**) by Sex, 2005–2013

Figures 4, 5 and 6 show DS prevalence by AHFS categories, year, and sex. Overall prescription prevalence (Fig. 4a) was about 10 times higher among women compared to men but there was little change over the study period for either sex. Multivitamin preparations also changed little over time (Fig. 4b) and prevalence was over 50 times higher among women. Prenatal vitamins accounted for 87% of all multivitamin prescriptions. If prenatal vitamins were not included, multivitamin prevalence was 3.1 times higher among women (10.8 vs. 3.5 SMs/1000 SMs for entire study period). For iron preparations (Fig. 5a), women had about 30 to over 50 times the prevalence of men (depending on year). There was little change over time in iron preparation prevalence for men, but women showed a decline (78 to 65 SMs/1000 SMs) over the study period. Replacement preparation prevalence (Fig. 5b) changed little among men but women showed an increase in prevalence in the 2006 to 2009 period followed by a decline. Antacid/absorbent prevalence declined throughout the study period for women and men also showed a very slight decline (Fig. 5c). Cathartics/laxative prevalence declined after 2008 returning to near 2005 levels by 2012 among men but not women (Fig. 5d).

For the AHFS categories involving vitamins (Fig. 6), trends were similar for men and women with higher prevalence among women for all AHFS categories. Vitamin B/B Complex (Fig. 6a) and Vitamin E (Fig. 6d) prevalence declined, while Vitamin C (Fig. 6b) and Vitamin D (Fig. 6c) prevalence generally increased.

### Temporal changes by age

**Fig. 7:**
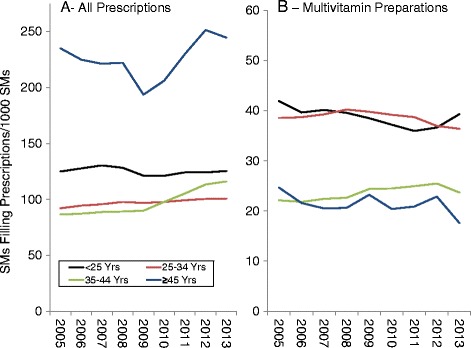
Prevalence of Prescriptions Filled for All Prescriptions (**a**) and Multivitamin Preparations (**b**) by Age, 2005–2013

**Fig. 8:**
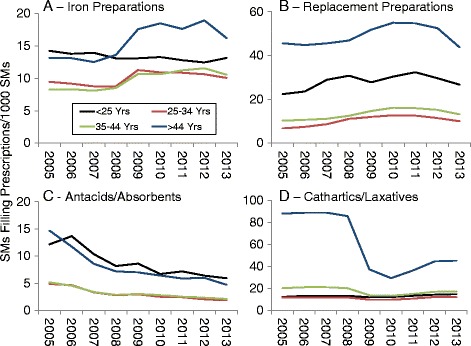
Prevalence of Prescriptions Filled for Iron Preparations (**a**), Replacement Preparations (**b**), Antacids/Absorbents (**c**) and Cathartics/Laxatives (**d**) by Age, 2005–2013

**Fig. 9:**
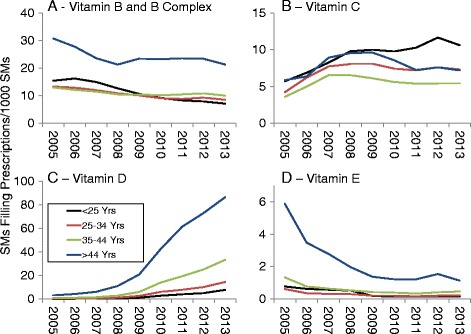
Prevalence of Prescriptions Filled for Vitamin B and B Complex (**a**), Vitamin C (**b**), Vitamin D (**c**), and Vitamin E (**d**) by Age, 2005–2013

Figures 7, 8 and 9 present DS prevalence by AHFS categories, year, and age. For all prescriptions (Fig. 7a), the highest prevalence was for the oldest age group (≥45 years). For multivitamin preparations (Fig. 7b) the two youngest age groups had the highest prevalence, almost 2-fold higher that the two older age groups. However, when only non-prenatal vitamins were considered, the prevalence for the entire study period was 4.7, 3.4, 5.0, and 13.7 SMs/1000 SMs for the <25, 25–34, 35–44 and ≥45 year olds, respectively, with very little change in prevalence over the time period (yearly data not shown).

For iron preparations (Fig. 8a), all age groups except the youngest demonstrated an increase in prevalence in the 2008 to 2009 period with only minor changes after this. Prescriptions filled for replacement preparations (Fig. 8b) generally increased in prevalence over the period with the highest prevalence in the oldest age group. Antacids/absorbents (Fig. 8c) showed a steady decrease in prevalence over the study period for all age groups with the highest prevalence maintained by the youngest and oldest age groups throughout the period. There was little change in the prevalence of cathartic/laxatives (Fig. 8d) for the two youngest age groups but the two older age groups showed declines from 2008 to 2010 with much larger declines in the oldest age group. Psyllium and sodium/potassium compounds accounted for 73% of cathartic/laxative prescriptions for those ≥45 year olds. The trends in prescriptions filled for cathartic/laxatives in the two older age groups were primarily due to temporal declines in the prevalence of sodium/potassium compounds with little change in psyllium. In those >45 years of age, the prevalence of sodium/potassium compounds declined from 59 SMs/1000 in 2008 to 5 SMs/1000 in 2010.

Vitamin B and B Complex prevalence (Fig. 9a) declined for all age groups during the study period. Vitamin C prevalence (Fig. 9b) increased for all age groups between 2005 and 2007–2009 and showed only minor changes after that. Vitamin D prevalence (Fig. 9c) rose in all age groups over the surveillance period while Vitamin E prevalence (Fig. 9d) decreased in all age groups. For VitaminB/B Complex, Vitamin D and Vitamin E, the largest temporal declines were for the oldest age group.

### Replacement preparations and Vitamin B and B complex

**Fig. 10:**
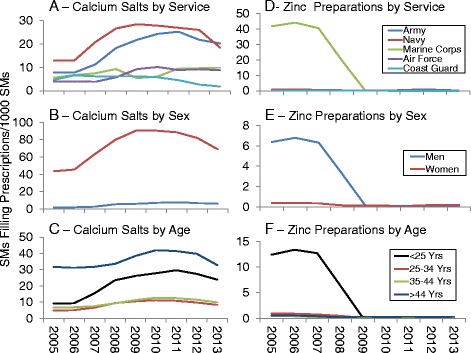
Prevalence of Prescriptions Filled for Calcium Salts by Military Service (**a**), Sex (**b**), and Age (**c**); Prevalence of Prescriptions Filled for Zinc Preparations by Military Service (**d**), Sex (**e**) and Age (**f**), 2005–2013

Further examination of replacement preparations showed that most prescriptions were for calcium salts (74%) and zinc preparations (12%). For calcium salts (Fig. 10a through 10c), the largest changes in prevalence occurred primarily in the Navy and Army, among women, and among those in the youngest and oldest age groups. For zinc preparations, the temporal prevalence declines were primarily among the youngest (<25 year) male Marines (Fig. 10d through 10f).

**Fig. 11:**
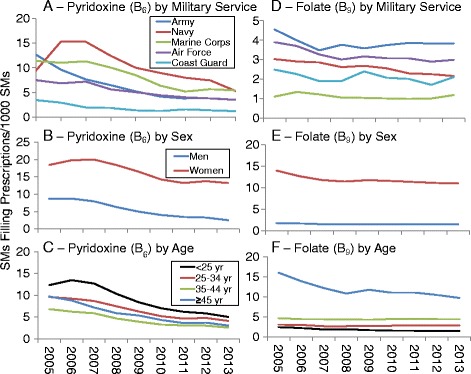
Prevalence of Prescriptions Filled for Pyridoxine (Vitamin B_6_) by Military Service (**a**), Sex (**b**), and Age (**c**); Prevalence of Prescriptions filled for Folate (Vitamin B_9_) by Military Service (**d**), Sex (**e**) and Age (**f**), 2005–2013

For Vitamin B and B Complex, most prescriptions were for pyridoxine (62%) and folate (27%). During the study period, pyridoxine showed prevalence declines for all military services, in both men and women, and for all age groups (Fig. 11a through 11c). For folate, there were prevalence declines in all services over the survey period expect the Marines. These declines were larger for women and those in the oldest age group (Fig. 11d through 11f).

## Discussion

This is the first investigation to describe, based directly on pharmacy records, demographic factors associated with temporal trends in the prevalence of oral DS prescriptions filled by the entire population of SMs. The most notable findings were 1) a generally greater prevalence of prescriptions filled by the oldest (>45 years) SMs for many AHFS categories; 2) greater prevalence of prescriptions filled by women for all AHFS categories; 3) a temporal decline in total prescriptions filled by Marine Corps personnel, accounted for largely by a decline in the prevalence of zinc preparations by younger male Marines; 4) a temporal decline in the prevalence of iron preparations filled by women; 5) a temporal increase in the prevalence of prescriptions for replacement preparations filled by women accounted for largely by calcium compounds; and 6) a temporal decline in the prevalence of prescriptions for cathartics/laxatives in the oldest age group (>45 years) accounted for largely by sodium/potassium compounds. Other temporal trends seen across virtually all demographic categories included: 1) a decline in the prevalence of prescriptions filled for antacids/absorbents; 2) a decline in prevalence of prescriptions filled for Vitamin B and B Complex, accounted largely by a decline in pyridoxine (Vitamin B_6_) prescriptions; 3) a decline in the prevalence of prescriptions filled for Vitamin E; and 4) an increase in the prevalence of prescriptions filled for Vitamin D.

The generally greater prevalence of filled prescriptions for many AHFS categories among the oldest SMs may be due to greater health care demands of these individuals. For most major diagnostic categories, ambulatory visits are greater among older SMs compared to younger ones [22]. This could lead to a greater number of prescriptions for treatment of specific medical conditions. In the civilian population, individuals with medical conditions are more likely to use DSs [12]. In the present study, women were also much more likely to fill prescriptions for all categories of DSs than men even after eliminating prenatal vitamins. In both military and civilian samples, women are more likely to consume over-the-counter DSs compared to their male peers ([8, 11, 23] Kennedy, 2013 #4478). Sex differences may be associated with psychosocial factors relating to greater health awareness in women. Numerous studies have shown that compared to men, women are more active consumers of medical care [24–26] and are generally more likely to make lifestyle changes in an effort to improve their health [27, 28].

Another study [16] noted a dramatic decline in the use of zinc preparations among male users of military pharmacies over a 5-year period from 2007 to 2011. That study [16] combined active duty Navy and Marine Corps personnel as well as Navy and Marine dependents for the purposes of analysis. The present study found the decline occurred primarily in young, active duty Marine Corps personnel, with Navy personnel showing little change. The reason for the high use and decline from 2007–2009 among young Marine Corps personnel is not clear but one possible reason may relate to findings from early studies suggesting that zinc preparations may reduce the symptoms and duration of acute respiratory illness [29] and the lack of an adenovirus vaccine in the 1999 to 2011 period [30]. Adenovirus is an infectious disease and a frequent cause of upper respiratory disease and pneumonia in military recruits and deployed SMs [30, 31]. A vaccine for this disease was developed in 1971 and was routinely administered to all military trainees. However, the manufacturer ceased production of the vaccine in 1996 and by 1999 adenovirus vaccine stores were exhausted [32]. A new manufacturer began producing the vaccine and by 2011 it was reintroduced into military training centers [32, 33]. There is strong evidence acute respiratory illnesses increased when the vaccine was not available and sharply decreased after it was reintroduced [33]. Zinc preparations may have been provided to Marine Corps recruits and others because prior to 2008 evidence suggested zinc preparations could reduce the duration and symptoms of respiratory illnesses [29]. However, shortly after 2007 studies were published indicating they may not be effective [34]. A Cochrane review of 18 trials indicated that oral zinc administered within 24 h of symptom onset may slightly shorten the duration of the respiratory illness in healthy people. The review found no association between oral zinc supplements and symptom severity, and the prevalence of minor adverse effects with zinc lozenges was high [35].

Compared to men, the markedly higher prevalence of iron prescriptions filled by women (30 to over 50 times higher) may be associated with the higher level of iron deficiency among women. Iron deficiency is defined as a serum ferritin of <15 μg/l. Using this criteria, the National Health and Nutrition Examination Survey found that about 14% of women and <1% of men were iron deficient [36], and this may have led to medical care providers offering, or women requesting, more iron-related prescriptions. Of importance for the military, studies have shown that iron supplementation can generally improve the aerobic capacity and physical performance among women of reproductive age, especially those that are iron deficient [37, 38].

There was a 17% decline (78 to 65 SMs/1000) in iron preparation prevalence among women during the study period. Routine iron supplementation is often recommended for pregnant women [39, 40] but in the late 1990’s differing opinions were published by several national organizations regarding the validity of this recommendation [40]. During the study period, studies, reviews, and editorials were published with conflicting information on the effectiveness of, and need for, iron supplementation among pregnant women [41–44] that may have led to some confusion among providers. The latest review for the US Preventive Services Task Force concluded that even for iron deficient women, the evidence is still inconclusive as to whether or not routine iron supplementation improves maternal or infant health outcomes, although it does appear to improve maternal hematological indices [45]. The birth rate in military women was relatively consistent in the 2005 to 2012 period [46, 47] so it is unlikely that reduced fertility (i.e., a reduction in the number of women who might be recommended iron supplementation during pregnancy) could account for the decline in iron preparation prescriptions. It is not clear what may account for the decline in iron preparations among women during the study period.

Another interesting observation was the temporal increase (followed by a decrease) in the prevalence of prescriptions filled by women for replacement preparations. Replacement preparations are a very broad category of substances designed to treat a variety of specific nutritional deficiencies. In the present study, 74% of replacement preparations involved calcium salts and of these, 64% were combined with some form of Vitamin D. Studies conducted shortly before or during the study period indicated that either the dietary intake of Vitamin D or the plasma level of 25-hydroxyvitamin D (25(OH)D) (the accepted clinical indicator of Vitamin D status) of Americans was insufficient [48–50]. Considerable attention was given to this fact in both the popular press [51–53] and in scientific/medical journals [54–56]. This information, combined with more accurate methods of measuring 25(OH)D [57] led to an increase in the recommended daily allowance of Vitamin D and calcium [58]. This may at least partly account for the increase over the study period in prescriptions filled for calcium salt preparations, especially those containing Vitamin D.

The decreasing temporal trend in cathartics/laxatives in those >44 years of age was accounted for largely by a decline in prescriptions filled for sodium/potassium compounds. In the 1990’s and into the 2000’s there were a number of case reports on patients who developed acute renal failure after the use of sodium/potassium compounds as a cathartic agent [59–61]. This was followed in 2005 by a case series of acute renal failure in 21 patients using these compounds over a 5 year period [62]. In December 2008 the US FDA issued a safety alert that documented 20 cases of kidney injury reported to the FDA and required new safety measures for drugs of this type [63]. The rapid decline in use by older SMs may have been associated with the potential for kidney injuries, the FDA warning, and the availability of other substances that could be used as cathartic agents.

Certain trends were seen across virtually all demographic categories and we attempted to explain these in our previous paper [18]. The decline in the prevalence of antacids/absorbents was likely due to the development and use of advanced drugs like proton pump inhibitors, potassium–competitive acid blockers and other drugs for treating gastroesophageal reflux disease that reduced the need for antacids/absorbents [64, 65]. The increases in Vitamin D prevalence was discussed above and was likely associated with better availability and more accurate assay procedures for determining 25-OH-D [57], knowledge that large portions of individuals may be Vitamin D deficient [48–50], and changes in national policy increasing the recommended daily allowance of Vitamin D [58]. Declines in the prevalence of Vitamin E may be associated with studies suggesting that high-dose Vitamin E increases mortality [66–68], results of large randomized prospective cohort trials suggesting few benefits for treating specific diseases [69–71], and recommendations of the US Preventive Services Task Force against the use of Vitamin E for primary prevention of specific diseases [72, 73]. The decline in B Complex Vitamin prevalence was accounted largely by a decline in pyridoxine (Vitamin B_6_) prevalence, and to a much lesser extent by the decline in folate (Vitamin B_9_) prevalence. Although pyridoxine had been suggested for the treatment of carpel tunnel syndrome, hyperhomocystemia, and other conditions [74–79], studies conducted over the study period generally did not support these uses [80–82], thereby possibly accounting for the decline in prevalence.

## Conclusions

Our previous work [18] described overall trends in prescribed oral DSs filled by all military SMs from 2005 to 2013. In the present paper, we examined patterns of DS prescriptions that were filled by specific demographic groups. Age, sex, and service-specific factors assisted in interpreting some temporal trends, while others were independent of these factors and seen across all demographic groups examined. The perceptions and knowledge of providers and patients appeared to be important for interpretation of trends in prescriptions and will continue to evolve as new knowledge emerges. Continuing surveillance of prescribed DSs will identify if the demographic trends observed here continue or if new patterns emerge.

## Abbreviations

AHFS: American hospital formulary system
DS: Dietary supplement
GC3: Generic Class 3
NDC: National drug code
PDTS: Pharmacy data transaction system
PEC: Pharmacoeconomic center
SM: Service member
US: United States.
